# Animal models for the risk assessment of viral pandemic potential

**DOI:** 10.1186/s42826-020-00040-6

**Published:** 2020-04-22

**Authors:** Mee Sook Park, Jin Il Kim, Joon-Yong Bae, Man-Seong Park

**Affiliations:** Department of Microbiology, Institute for Viral Diseases, College of Medicine, Korea University, Seoul, Republic of Korea 02841

**Keywords:** Animal model, Pandemic, Pathogenicity, Transmission, Virus, Zoonosis

## Abstract

Pandemics affect human lives severely and globally. Experience predicts that there will be a pandemic for sure although the time is unknown. When a viral epidemic breaks out, assessing its pandemic risk is an important part of the process that characterizes genomic property, viral pathogenicity, transmission in animal model, and so forth. In this review, we intend to figure out how a pandemic may occur by looking into the past influenza pandemic events. We discuss interpretations of the experimental evidences resulted from animal model studies and extend implications of viral pandemic potentials and ingredients to emerging viral epidemics. Focusing on the pandemic potential of viral infectious diseases, we suggest what should be assessed to prevent global catastrophes from influenza virus, Middle East respiratory syndrome coronavirus, dengue and Zika viruses.

## Introduction

Of the four types of influenza viruses, influenza A virus (IAV) and influenza B virus (IBV) cause major respiratory diseases to humans [1, 2]. The IAVs can be classified into different subtypes by the antigenicity of surface glycoproteins, hemagglutinin(HA) and NA(neuraminidase). So far, 18 and 11 subtypes have been identified from the HA and NA proteins, respectively, and the last two subtypes (17 and 18 subtypes in HA and 10 and 11 subtypes in NA) were recently discovered from bats [3, 4]. All other subtypes (H1 through H16 and N1 through N9) have been identified in aquatic birds, which are considered as the main reservoirs of IAVs [5]. In contrast to the IAVs, IBVs are classified into two antigenically distinct lineages, namely Victoria and Yamagata [1, 5, 6]. While the IAVs infect diverse avian and mammalian hosts including humans, the IBVs are circulating mostly among human beings with a few exceptions of spillover cases reported in seals and swine [7–10]. IAV and IBV infections show similar clinical signs of ‘influenza-like illness’ and outcomes [11–14].

There have been four major influenza pandemics since 1918 with some glimpses of pandemic-like events in history [15–17]. The H1N1 influenza pandemic of 1918 (pdm1918) is estimated to have caused up to 50 million human deaths across the globe [18], symbolizing how devastating one pandemic outbreak can be. It is believed that influenza pandemics can be occurred by antigenic shift, which generally results from the introduction of certain gene segment(s) from nonhuman sources to human infecting IAVs through a genetic reassortment process [5, 16]. The efficient human-to-human transmission and lack of immunity against the novel virus in humans can be driving forces to facilitate the dissemination of the virus and then to result in a pandemic. After a pandemic wave, the virus may lose momentum under increasing immune pressures among humans and persist as a seasonal virus. This seasonal virus will retain genetic mutations by circulating season by season, and its viral antigenicity may change, which is so-called antigenic drift, and it is the main reason that the vaccine viruses need updates every year. Currently, the H1N1 and H3N2 subtypes of IAVs, which are the descendants of 2009 and 1968 influenza pandemics, respectively, and the Victoria and Yamagata lineages of IBVs are circulating as seasonal viruses in humans.

Before the H1N1 pandemic in 2009 (pdm2009), an avian H5N1 IAV had been remarked as a strong candidate that would cause a next pandemic given accumulating human infection cases with the virus [19, 20]. Recently, an avian H7N9 virus has become the focus of attention concerning the increasing number of human infection cases in China [21, 22]. However, it is important to remember that pdm2009 was caused unexpectedly by a swine origin IAV [16], emphasizing the importance of the surveillance of swine IAVs [23]. There are also other subtypes of avian HA and NA isolated from human influenza cases sporadically [24, 25]. Given their pandemic potential, we need to assess these human-infecting zoonotic IAVs in detail by comparing with the viruses that had caused past influenza pandemics.

Recently, Middle East respiratory syndrome coronavirus (MERS-CoV) is dubbed ‘camel-flu’ virus [26]. Seven years after its first human infection in 2012 [27–29], more than 2400 human cases have been reported with approximately 35% case fatality rate [30]. MERS-CoV has a single-stranded positive-sense RNA genome consisting of two partially overlapping large replicase open reading frames (ORFs) and at least nine downstream ORFs including the ORFs encoding the four canonical structural proteins of coronaviruses, the envelope proteins S, E, and M and the N protein [31]. Similarity of MERS-CoV with influenza viruses is not in its genome organization but probably in its respiratory symptoms, zoonotic potential, and the mode of respiratory transmission [32–34]. In addition to influenza viruses and MERS-CoVs, arthropod-borne viruses, such as dengue and Zika, may also pose pandemic threats even though persistent human-to-human transmissions have been rarely reported [35–39]. In this review, we intend to figure out the recipe and the ingredients of a pandemic by looking into the past pandemic events.

## Main text

### Zoonotic origins of influenza pandemics

**Fig. 1:**
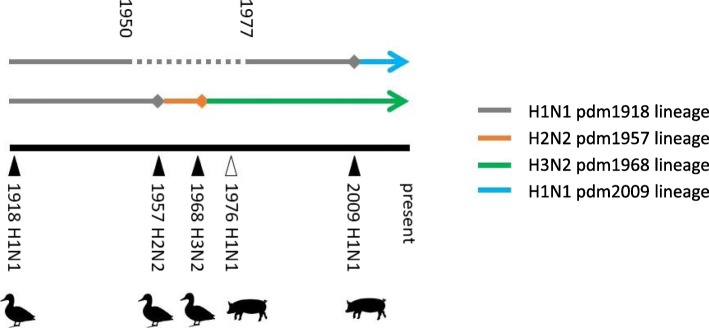
Timeline of influenza pandemics. Each pandemic event is marked by a bold arrowhead on the timeline, which is not to the scale. The open arrowhead designates the ‘aborted pandemic’. The animal symbols designate the host origins of the HAs of the pandemic strains. Lines with the diamond heads designate the duration and the end of the circulation. The arrowed lines designate being circulated currently. The dotted lines designate unknown

IAVs have eight segmented genomes of single-stranded, negative-sense RNAs, which express similar major proteins, such as polymerase basic 2 (PB2), polymerase basic 1 (PB1), polymerase acidic (PA), HA, nucleoprotein (NP), NA, matrix 1 (M1) and matrix 2 (M2), nonstructural 1 (NS1), and nonstructural 2 (NS2/NEP) [1]. Studying the past influenza pandemics helps us to understand the mechanisms of such devastating outcomes. Theoretically,144 different IAV subtype viruses can be generated by the combinations of 16 HA and 9 NA subtypes of avian IAVs. However, only the H1N1, H2N2, and H3N2 subtypes have been identified as the causes of human influenza pandemics. Of these, the H1N1 subtype caused the 1918 and 2009 pandemics and the ‘abortive pandemic’-like swine influenza epidemic in 1976, which hundreds of soldiers at Fort Dix, New Jersey, the United States were infected with [15, 40, 41] (Fig. 1). Although diverse IAV subtypes have been isolated from swine, H1, H2 and H3, and N1 and N2 subtypes have been mainly established [42–44]. As mentioned above, the pdm2009 and 1976 H1N1 viruses were swine-origin and readily transmissible among humans [15, 16, 45]. Another pandemic H1N1 virus, pdm1918, however, appeared to be closely related with avian strains, which would be ultimately the ancestor of subsequent human and swine H1N1 IAVs [46]. Since human-infecting avian IAVs were shown to be less transmissible among humans [47–49], a ‘spill-over’ infection with avian IAVs was not likely to be a direct source of pdm1918 [50, 51]. As the receptor specificity is considered another prerequisite for avian IAVs to infect and transmit to humans, there are some doubts about the avian-origin pdm1918 [52]. The H2 HA of the 1957 H2N2 pandemic and the H3 HA of the 1968 H3N2 pandemic were introduced from the avian reservoir to circulating human IAVs [53, 54], but whether the reassortment events occurred in humans or in other hosts, such as swine, immediately before transmitting to humans remains unanswered [16].

### Swine as an adaptation host of IAVs

IAVs can be largely divided into the human-like and avian-like types given the receptor specificity of their HA proteins. Normally, human-like IAVs bind to α-2,6 sialic acid (α-2,6 SA) receptor whereas avian-like IAVs prefer α-2,3 SA receptor [55, 56]. It has been revealed that an aquatic bird mallard expresses more α-2,3 SA than α-2,6 SA in the respiratory tract but α-2,6 SA is barely expressed in the intestinal tracts [57]. The preference of avian IAVs to α-2,3 SA might be related with fecal transmission of the viruses [58, 59]. Regarding zoonotic transmission of IAVs, swine is considered the intermediate host that can shuffle genetic segments between avian and human IAVs to produce a novel strain [54]. Because swine expresses both α-2,3 and α-2,6 SAs in the upper respiratory tract, avian and human IAVs all can infect swine [60–62]. It has been also challenged by additional studies that demonstrated similar SA distribution between human and swine [63–66]. However, it has been shown that avian IAVs could transmit between swine, and novel strains could be generated from contact swine by the genetic reassortment between avian and swine IAVs [67], which cannot be demonstrated in humans. Hence, it appears that swine rather than humans may play a major role for the generation of novel strains at the interface of avian and human IAVs [68–70], and swine might be considered an adaptation host of IAVs, as indicated in the cases of zoonosis [71–76] and reverse zoonosis of IAVs [43, 77]. Then, it should be questioned whether avian IAVs can be transformed into a pandemic virus by the adaptation only in humans. Even though some reports indicated acquired transmissibility of avian IAVs through multiple passages in ferrets [78] and transmissibility of avian IAVs in swine [67], it may be limited contact opportunities of the same avian IAV to be repeatedly adapted in humans, as demonstrated in Herfst et al. [78]. Whether a rare adaptive mutant would grow out to dominate in the human host would be another issue. Unlike severe symptoms observed in novel avian IAV-infected patients, however, swine may be asymptomatic when infected with avian IAVs [79, 80]. Unless efficient transmissibility of an avian IAV in humans was adaptively acquired during a single human infection, there would be only very limited close contact transmission from the patient to the care giver. Close contact transmission of avian H7N9 IAVs between patients and care givers have been recognized, but the contacted care givers have rarely shown the signs of infection [81–83]. This may demonstrate why avian IAVs have acquired necessary adaptive mutations in swine rather than in humans to be pandemic viruses [69].

### Ingredients and recipe of influenza pandemics

**Fig. 2:**
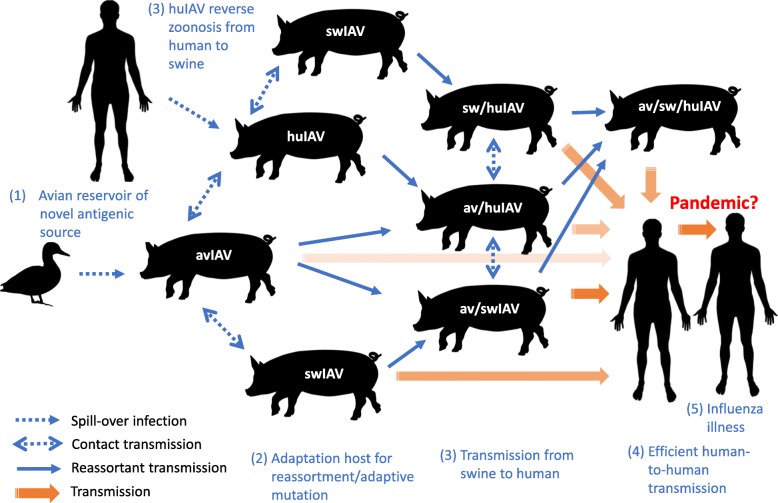
The ingredients and recipe of influenza virus pandemic. The workings of the five ingredients of IAV pandemics are depicted. The ingredients are numbered (1) through (5). Avian IAV, swine IAV, and human IAV are given as avIAV, swIAV and huIAV, respectively. The viruses generated through (1), (2), and (3) may have a diverse range of transmissibility. When a virus with a nonhuman origin HA and an efficient human transmissibility gets transmitted from the adaptation host swine to human (4), a pandemic might ensue (5)

From the examples of the past influenza pandemics discussed so far, the recipe of a pandemic may be drawn up. The ingredients of influenza pandemics appear to be (1) non-human animal reservoir(s) that provides novel antigenic sources continuously, (2) adaptation host(s) where accumulated mutations result in host specificity changes or genetic reassortment occurs, (3) proper transmissibility between adaptation host(s) and humans back and forth, (4) efficient human-to-human transmission, and (5) pathogenicity in humans (Fig. 2). The second and third ingredients may work together to generate a virus with the human adapted genes with a new antigenic flavor. We have seen that the influenza pandemics came about with these ingredients and the human activity of socializing and traveling. Determining whether a virus has these ingredients might be an important step in assessing its pandemic potential.

### Influenza epidemics

The sudden reappearance of 1950s seasonal H1N1 strains in 1977 that was dubbed ‘Russian flu’ right after the 1976 swine IAV epidemic spurred the awareness of the need for pandemic planning [15, 40]. Although the virus had rapidly spread among people under 25 years of age and had been ‘drifting’ as seasonal strains until the appearance of the pdm2009 [18], the 1977 H1N1 virus may not be considered a pandemic virus. The virus was only a reappearance of previous human IAV most likely by an accidental release and met an immunity gap among young people. The virus was not of a nonhuman reservoir origin, according to the pandemic ingredients summarized above. In case of the H5N1 and H7N9 avian IAVs, they may lack efficient transmissibility to and between humans. However, as shown in ferret studies, they should be under close surveillance for their pandemic potential in advance. In addition to these avian IAVs, swine IAVs should be also monitored because novel strains can be generated by genetic reassortment in swine between avian and human IAVs, as presented in the genesis of pdm2009. In contrast to IAVs, IBVs do not have animal reservoirs, so they might be considered a virus of less pandemic potential.

### MERS-CoV

MERS-CoV appeared to originate from a bat coronavirus [28, 84–87] and has become enzootic since a certain time point among dromedary camels [32, 88–90], which is readily transmissible to and between humans [34]. Unlike other human virus, such as measles [91], MERS-CoV might evolve constantly in the dromedary camels [92], which show a high rate of seroconversion and carry the virus mostly asymptomatically [90]. Continuous back and forth transmission between humans and the dromedary camels constitutes very similar situations with IAVs. Frequent recombination during MERS-CoV replication in the reservoir host [93–95] might be used as a tool of adaptation by antigenically novel MERS-CoV strains or closely related bat coronaviruses, similarly as genetic reassortments of IAVs in swine [96]. If there might be a MERS-CoV pandemic, subsequent MERS-CoV epidemics by antigenically ‘drifted’ strains might follow the pattern of seasonal influenza viruses until antigenically shifted (recombined) MERS-CoV strains hit humans again.

### Dengue and Zika viruses

Mosquitos are a vector and non-human reservoir of dengue and Zika viruses. Back and forth transmission of these viruses between mosquitos and humans and the ‘antibody dependent enhancement’ of infection to dengue and Zika viruses might potentially support the expansion of susceptible human pools [97–99]. However, currently there are very limited cases of human-to-human transmission of these viruses through body fluid contacts [35–39], and it is unlikely to result in rapid global spreading of the viruses like IAVs, especially since mosquito distribution is geographically limited [100]. Hence, possible human-to-human transmission of the dengue and Zika viruses is inevitably limited by the requirement of intimate contacts or blood transfusion [101]. Even though this ecological limitedness, solid control measures against mosquitos should be implemented to prevent dissemination of these arthropod-borne viral diseases in a global scale.

### Animal models

Small laboratory animals are surrogate models used in the experiments of human infecting viruses. Viral behaviors in a natural host are often different from those in humans. Viruses causing serious diseases in humans are often asymptomatic in their natural hosts. This is why natural hosts have a limitation as animal models. In case of IAVs, avian and swine species should be considered the natural reservoir animals, and in case of MERS-CoVs, bats and dromedary camels [32, 87, 90]. Viruses causing severe diseases and deaths in humans are the targets of prevention and control. Researches using animal models for such viruses are carried out in two main directions: (1) investigation of viral characteristics in hosts, such as replication capacity, cellular tropisms, pathogenicity, and transmission, and (2) development of antivirals and vaccines. No animal models can be a perfect replicate of humans. Certain animal models can have advantages in representing viral infection and, at the same time, disadvantages in other aspects. Therefore, experimental questions may determine the best animal model, and experiments should be conducted such a way that the results obtained using animal models can be translated to humans.

### Animal models of influenza viruses

Non-reservoir animals used for influenza virus infection experiments include ferrets, mice, guinea pigs, Syrian hamsters, and non-human primates [102–109]. Historically, the discovery of the first human influenza virus was made by infecting ferrets with throat washings of influenza patients [110]. Ferrets could also be readily infected with swine influenza virus. The different species of guinea pigs, mice, rabbits, hamsters, hedgehogs, and monkeys did not develop flu-like symptoms [103]. However, these asymptomatic animals have become useful for special purposes of influenza virus infection experiments now because virus detection and titration methods have become sophisticated (Table 1). Especially, mice and guinea pigs are the most accessible animal models cost- and space-wise. In the case of mice, human infecting influenza viruses do not usually infect mice well, which is overcome by adapting the virus in mice through serial passages [103, 119, 120]. Mice express the avian type α-2,3 SA in the lower respiratory tract, similarly as humans, but not the human type α-2,6 SA [121]. This is in line with the tendency of experimentally inoculated avian IAVs, regardless of low-pathogenic (LPAIV) or highly pathogenic avian IAVs (HPAIV), being mouse lethal with a relatively low 50% mouse lethal dose (MLD_50_) [104, 122]. As far as seasonal isolates of human IAVs are concerned, DBA/2 mice have been shown to be highly susceptible to diverse strains of un-adapted IAVs [123, 124]. Ferrets, guinea pigs, and Syrian hamsters could be infected with most of IAVs without adaptation, but only guinea pigs could be infected with IBV without adaptation and supported the airborne transmission [108, 111]. Syrian hamster has been tried to replace mice because IAVs could infect it without adaptation and there were airborne transmissions among Syrian hamsters [108]. However, since guinea pigs can be infected with un-adapted IBVs as well as IAVs and support airborne transmissions, which is readily detectable by the nasal wash titer, any advantage of Syrian hamsters over guinea pigs awaits further reports of the use of animal models in influenza virus research. Nonhuman primates (NHPs) are genetically closest to humans. Although NHPs are not a readily accessible animal model, NHPs are indispensable in the cases of vaccine and antiviral tests, where data relevant to humans in terms of pharmacokinetics and physiology are critical [125]. ‘Animal Efficacy Rule’ of the United States Food and Drug Administration (FDA) requires for the therapeutics to demonstrate efficacy in two animal models manifesting human-like symptoms including at least one non-rodent model [126]. Although the needs of testing in NHP models are clearly present [113–117], most laboratories cannot afford NHPs, and there is other ethical uneasiness about using NHPs. How best to do without NHPs may be a persisting issue in search of appropriate animal models. In the case of influenza virus research, among the frequently used animal models, ferrets appear to be the only non-rodent model other than NHPs [127].

**Table 1:** Animal models for influenza virus

Animal model	Model for			References
	Pathogenicity	Transmission	Antiviral	
Ferret	O	O	O	[103, 104]
Mouse	O	?	O	[103, 104]
Guinea pig	?	O	?	[103, 104, 111, 112]
Syrian hamster	?	?	?	[104, 108]
Nonhuman primate	O	?	O	[104, 113–118]

The designation of “O” means that there are many studies using the animal for the purposed study

The designation of “?” means that there are no or not many studies using the animal for the purposed study

### Animal models of MERS-CoV infection

Animal models of MERS-CoV are restricted by the availability of the receptor DPP4 that contains distinct amino acid sequence motif. Besides the reservoir host dromedary camels and bats, NHPs, rabbits, and other livestock animals, such as goat, cow, sheep, and pig, have been shown to express DPP4 that can bind to MERS-CoV [85, 128–130]. However, DPP4 of frequently used small animal models like mouse, hamster, and ferret did not bind to MERS-CoV [128]. In NHPs, MERS-CoV infection showed similar clinical signs as in humans, ranging from mild to severe, depending on the species of NHPs. Although DPP4 expressions were similar between rhesus macaques and common marmosets, the disease severity was from mild to moderate and from moderate to severe, respectively [131, 132]. Lack of replication of MERS-CoV in small animal models poses problems of cost and space, especially since experiments using MERS-CoV must be carried out in an animal biosafety level 3 facility [29, 133–135]. Mouse engineering technology has been deployed in diverse ways to generate mice expressing human type DPP4 (hDPP4) [136–140]. MERS-CoV infection in mice having hDPP4 exhibited only moderate signs of respiratory pathology, most likely due to the low level expression of hDPP4 in the mouse lung [135], but MERS-CoV could be adapted in these mice to a more pathogenic virus [139, 141]. In addition to NHP and hDPP4-mouse models, rabbits might be a good candidate for MERS-CoV transmission experiments due to its camel-like receptor distribution in the upper respiratory tract (Table 2) [142, 150]. However, while dromedary camels and New World camelids could transmit MERS-CoV upon contact, rabbits could hardly transmit the virus [130, 143]. MERS-CoV has been shown to use α-2,3 SA as a receptor assistant, which dromedary camels but not rabbits express in the nasal epithelium [130, 151, 152]. Humans do not express the primary receptor DPP4 in the upper respiratory tract but transmits MERS-CoV well [153]. Despite the controversies, humans have been reported to express α-2,3 SA in the upper respiratory tract [61, 64]. Contribution of the ‘pre-attachment’ receptor α-2,3 SA or any other ‘assistant’ receptors to MERS-CoV transmission might be worth further investigation [151]. As far as the Animal Efficacy Rule of FDA is concerned, there appears no other choices but hDPP4-mouse and NHP models in the case of MERS-CoV studies. Human-like symptoms of MERS-CoV infection have not been reproduced in other animals than hDPP4-mice and NHPs.

**Table 2:** Animal models for MERS-CoV

Animal model	Model for			References
	Pathogenicity	Transmission	Antiviral	
hDPP4-mouse	O	?	O	[136–140]
Rabbit	?	?	?	[142–145]
Nonhuman primate	O	?	O	[131, 132, 134, 146–149]

The designations of “O” and “?” are the same as in Table 1

### Host determinants contributing to pandemic viruses

Starting from the distinct receptor specificities of the HA proteins between avian and human IAVs, host restriction determinants of IAVs have been documented [56]. Receptor specificity and amino acid signatures at PB2 residue 627 are well established host determinants critical for the interhost transmission of IAVs [154]. However, IAVs with avian or human receptor specificity can infect swine, as mentioned above. Furthermore, the PB2 protein of the triple reassortant swine IAV lineage, which comprises a majority of North American swine IAVs [155], retains the avian type E627 (glutamate in the PB2 residue 627) [156]. This was also a part of the molecular signatures of pdm2009 [16]. Some human infecting avian IAV isolates have shown acquisition of the human type K627 (lysine in the PB2 residue 627) but not acquisition of the human type receptor specificity determinants, and some acquisition of both [80, 157, 158]. IAVs were shown to bind cells lacking sialic acid, and replicated efficiently [159]. Acquisition of PB2 K627 might be more advantageous than acquisition of human type receptor specificity for avian IAVs to replicate in the upper respiratory tract of humans, which is not an optimal temperature for PB2 E627 [157]. It has been also shown that the PB2 E627K mutation can emerge in a human case infected with an avian IAV [160]. Of note is that, although there have been avian-to-human transmission cases of avian H5N1 and H7N9 IAVs, there have been no sustained human-to-human transmission of avian IAVs. Indeed, it has been reported that an H5N1 HPAIV harboring the human-type PB2 E672K mutation (change from glutamate to lysine in the position 627) and human-type HA Q226L and G228S mutations (change from glutamine to leucine and from glycine to serine in positions 226 and 228, respectively, by H3 numbering) by itself could not transmit via airborne droplets between ferrets [78]. This may suggest that airborne transmissibility of avian IAVs in humans might not be determined only by the presence or absence of molecular determinants. Several studies using reassortant viruses have shown that the competence of reassortant viruses may not be predicted simply by the presence of the specific molecular determinants [161–164]. An experiment testing a swine ‘mixing vessel’ hypothesis by co-housing pigs infected with an avian H1N1 strain or with a swine H3N2 strain and naive pigs revealed 40 and 60% transmission efficiency, respectively [67]. In that experiment, the reassortant viruses appeared to be well replicated (59/63) in the middle or lower respiratory tract, regardless of the presence of swine PB2 or avian PB2, although all but one reassortants (62/63) contained the swine HA. Four out of the 63 reassortants did replicate in the upper respiratory tract and three out of four of those were the swine PB2-containing reassortants [67]. What we can learn from this experiment is that the reassortant with an avian HA is not frequently selected in pigs and that the reassortant with swine PB2 is selected for the replication in the upper respiratory tract of pigs. The proportion of IAV infection in farmed pigs is relatively low [68], and the likelihood of co-existing of pigs infected with avian IAVs and/or with swine IAVs in the same pen may be even lower. However, avian and swine IAV reassortants have been established and isolated in pigs [155], which is the evidence of ongoing ‘genetic mixing’ of IAVs.

### From surveillance to determination of the pandemic potential of viruses

Surveillance of newly emerging IAVs may be approached in two ways; isolating and sequencing a virus using next-generation sequencing (NGS). As we discussed above, it is not enough to look for the molecular determinants by sequencing. Even though full genome sequences are recovered, and their evolutionary relationships are reconstructed, further studies using the viruses should be carried out [165, 166]. The classical method of growing viruses in Madin-Darby canine kidney (MDCK) and human airway epithelial A549 cells might be the first step after a genetic sequence analysis. The MDCK cells express both α-2,3 and α-2,6 SAs and support replication of influenza viruses ubiquitously due to the lack of the Mx protein anti-influenza signaling [167, 168]. Therefore, viral growth in the MDCK cells is considered to evaluate the inherent growth potential of the viruses under such a condition where no innate and adaptive immune responses of the host are counted in [157]. On the other hand, the A549 cells, expressing more α-2,6 SA than α-2,3 SA like in the human upper respiratory tract, may indicate the growth potential of the viruses in the human upper respiratory tract [169, 170]. Any swine isolates – avian, swine endemic, or reassortant origin – showing equivalent to or better growth rates in MDCK and A549 cells than the swine-origin pdm2009 virus may be further studied for their pathogenicity and transmissibility in animal models, to determine their pandemic potential.

### Viral pathogenicity and transmission in animal models as pandemic potential

Viral pathogenicity is related to host cell tropism but may be separate from the susceptibility of hosts to the virus. IAVs are pathogenic to humans but not guinea pigs, although both are permissive to the virus. The degree of pathogenicity, the virulence of an IAV, is inevitably associated with how well the virus replicates in a tissue or an organ, impairment of which results in a serious disease. In cases of respiratory viruses, those replicating in the lower respiratory tract tend to be more pathogenic than those in the upper respiratory tract [171, 172]. Therefore, viral pathogenicity is closely related to inherent replication ability and receptor specificity of the virus. An avian IAV with PB2 K627 has been shown to replicate better in both upper and lower respiratory tracts in mice and more pathogenic to the infected mice than with PB2 E627 [157]. However, in case of the pdm2009 isolate A/California/04/09 virus (CA04), the virus lacked previously identified molecular markers of IAV virulence or transmissibility [173], although was more pathogenic to mice than the seasonal H1N1 virus [174–176]. Droplet transmissibility of pdm2009 in ferrets was shown to be slightly lower than the seasonal H1N1 virus whereas their contact transmissibilities were equally efficient [174]. This suggests that antigenic novelty might play a more important role for the pandemic potential of a certain IAV than viral transmissibility.

### Mice as a pathogenicity indicator of IAVs

Ferrets have been known to have human-like glycan distributions in their respiratory tract and may develop respiratory symptom after IAV infection [104, 127]. However, although most human IAVs including swine IAVs are not pathogenic to mice, mice could be a good initial testing model in terms of cost and handling easiness compared with ferrets, especially for an isolate containing avian origin HAs. Since mice have α-2,3 SA in the lower respiratory tract [121], avian IAVs tend to be pathogenic to mice without a prior adaptation [177]. Therefore, viral pathogenicity in mice could be an initial pathogenicity indicator of certain IAVs. The first pdm2009 isolate CA04 was more pathogenic in BALB/c mice than the later pdm2009 isolates [176]. The pdm2009 isolates from fatal cases were also more pathogenic in mice than from the mild cases, and replacing the HA of the mild case isolate with that from the fatal case could make a more pathogenic virus in mice [178]. These experiments show that IAV pathogenicity in mice may reflect inherent lung pathogenicity of IAVs in humans. However, the results of mouse experiments should be interpreted in comparison with a pathologically well-characterized control.

### Human IAVs adapted in mice vs. avian IAVs adapted for human-to-human transmission

Through a serial adaptation process, more pathogenic viral isolates can be recovered. For an IAV with the receptor specificity to α-2,6 SA to replicate in mice, where there is hardly any or small amount of α-2,6 SA in the respiratory tract [121, 179, 180], the virus must have used surrogate receptors such as C-type lectins or else [181–183]. IAVs have been shown to bind to and replicate in SA-free or sialidase treated cells, although to a lower degree than in the untreated cells [159, 183]. Abolishing NA activity has been shown to be another mechanism of adapting to the host expressing a low level of the specific receptor [184]. Indeed, the HA protein of a mouse-adapted pdm2009 has been shown to have acquired higher affinity to α-2,3 SA and lower affinity to α-2,6 SA compared to the wild type [185]. The difference between a human IAV adapted in mouse and an avian IAV infecting humans may be determined by the usage and availability of appropriate SA or equivalent molecules. To be pathogenic to mice, human IAVs with α-2,6 SA specificity must adapt to use non-SA receptors or α-2,3 SA abundant in mice [180], but avian IAVs with α-2,3 SA specificity may have a possibility to replicate in the lower respiratory tract of humans without a prior adaptation.

### Conceptual design of human, swine, and ferret respiratory tracts

**Fig. 3:**
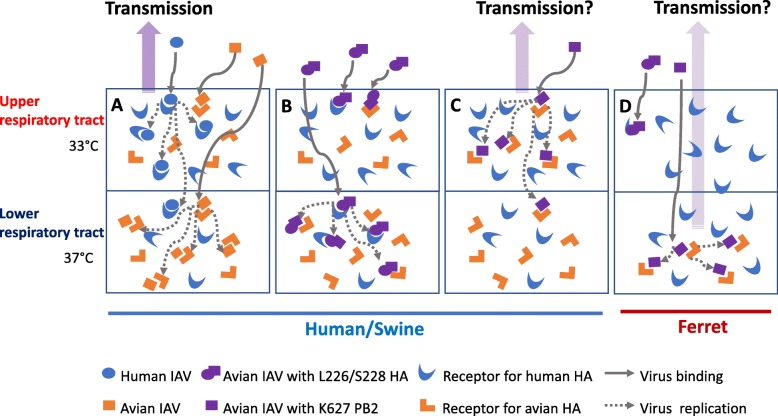
Conceptual construction of human, swine and ferret respiratory tract, IAV infection and transmission. The human type and avian type IAV receptor distributions are depicted in simplicity based on de Graaf et al. (2014) [63]. **a** Human and swine IAVs replicates primarily in the upper respiratory tract in the respective hosts and some may spread into the lower respiratory tract. The viruses are transmissible. A high dose of avian IAV may allow the virus to reach the lower respiratory tract for replication and some may overflow into the upper respiratory tract, but the receptor binding and internalization not leading to the virus replication may have the virus ‘cleaned up’. **b** A high dose of an avian IAV with the dual specificity mutations Q226L and G228S or Q226L alone may behave like an avian IAV in human or swine. **c** An avian IAV with E627K change in PB2 may replicate in the upper respiratory tract of human or swine and may spread into the lower respiratory tract like human or swine IAV in human or swine (**a**), and the virus is theoretically transmissible. **d** Human or swine IAV replication in ferrets may be similar as in human or swine. An avian IAV with the HA change for the avian and human dual receptor specificity may be trapped in the upper respiratory tract of ferret and may behave in ferret like in human or swine (**b**). Avian IAV with E627 or K627 PB2 might behave similarly in ferrets. An avian IAV, without the specific receptor in the upper respiratory tract of ferret, may replicate in the lower respiratory tract but relatively poorly due to relatively low expression of SAα-2,3 glycans. The overflowing avian IAV, although with a low likelihood, may not be ‘cleaned up’ due to lack of SAα-2,3 glycans in the upper respiratory tract of ferret, and may occasionally get transmitted to nearby ferrets

It has been known that humans express α-2,3 and α-2,6 SAs in the lower and upper respiratory tracts, respectively [61]. But, it has been also reported that humans express both α-2,3 and α-2,6 SAs at a similar level in the upper respiratory tract [64]. Given the availability of animal models that reflect human respiratory diseases, we conceptually suggest human, swine, and ferret respiratory tracts in Fig. 3, based on the study of de Graaf et al. [63]. Human or swine IAVs with α-2,6 SA specificity would be trapped in the upper respiratory tract of the respective host, where α-2,6 SA is abundant (Fig. 3a). Some replicating viruses may overflow down to the lower respiratory tract, where both α-2,3 and α-2,6 SAs are expressed. Avian IAVs with α-2,3 SA specificity would be also trapped in the upper respiratory tract by α-2,3 SA, but replicate poorly due to the unfavorable temperature. Under such circumstances, only a high dose of avian IAVs allows to escape the trapping in the upper respiratory tract and reach the lower respiratory tract, where the temperature is more favorable for avian IAVs. Even though avian IAVs might overflow from the lower respiratory tract up to the upper respiratory tract, the virus might not replicate there due to the unfavorable temperature, unless there was the PB2 E627K mutation. This may be the reason that avian IAVs are not easily transmissible between humans. In terms of viral adaptation, a rare appearance of avian IAV mutants with α-2,6 SA specificity may not have special selective growth advantages in the lower respiratory tract of humans due to the overwhelming dominance of α-2,3 SA. Only when avian IAVs replicating in the upper respiratory tract, although poorly, acquires the PB2 E627K mutation or a reassortment, with or without acquisition of α-2,6 SA specificity at the same time, the variants may grow out well [67]. Serial passaging of a wild-type H5N1 HPAIV in ferrets could not make the virus airborne transmissible between ferrets, but only those containing the mutations conferring the human type α-2,6 SA specificity and PB2 E627K could acquire airborne transmissibility after several passages in ferrets [78]. Basically, these experiments suggest that, even if a rare mutant retaining the human-type receptor specificity and PB2 determinants might appear during replication of avian IAVs, the mutant might not be easily selected to a domination over multiple passages, at least in ferrets. The reason may be that avian IAVs have their niche of efficient replication in the lower respiratory tract of human, swine, or ferret (Fig. 3). The issue of avian IAV adaptation in humans may not be whether adaptive mutations appears but whether there is a selective force enough for a virus to possess adaptive mutations to grow out to dominance.

### Experimental observation and conceptual transmission model

The HA protein of avian H5N1 IAVs has been reported to require both human-type Q226L and G228S mutations to bind to both α-2,3 and α-2,6 SAs [186], and that of avian H7N9 IAVs only needs the Q226L mutation [122, 187]. Interestingly, a H7N9 human isolate was contact transmissible in pigs, regardless of PB2 E627 or K627, but only in the acquisition of human-type HA Q226L determinant, which indicates the importance of the acquisition of human-type receptor specificity for the avian IAV transmission in pigs, potentially also in humans [188]. However, as demonstrated in Fig. 3b and c, the acquisition of human-type PB2 K627 would have been more critical due to the presence of SAα-2,3 glycans in the upper respiratory tract of pigs. Avian IAVs with the PB2 E627K mutation should be able to replicate in the upper respiratory tract of humans and may be transformed to be transmissible between humans (Fig. 3c). However, they were poorly transmissible in ferrets (Fig. 3d). There have been no reports that investigated the transmissiblity of avian IAVs with the PB2 E627K mutation between humans. All the first three human isolates of avian H7N9 IAVs, which contained the human-type PB2 E627K and HA Q226L mutations, showed approximately 30% airborne transmissibility in ferrets [48, 80, 122]. These appear to match with the conceptualized transmission in ferrets of Fig. 3d. The H7N9 isolates with PB2 K627 and HA L226 would have replicated in the upper respiratory tract of ferrets, but their replication might have been inefficient since the receptor binding of the H7N9 Q226L HA was still weaker to α-2,6 SA than to α-2,3 SA [187]. Airborne transmission of the H7N9 virus in ferrets lacking α-2,6 SA might be explained by the overflow of the virus replicating in the lower respiratory tract, since ferrets poorly express α-2,3 SA in the upper respiratory tract [189]. This model also indicates similar level of transmission of avian IAVs in ferrets without any human-type determinants. Therefore, the conceptual model of IAV transmission based on the receptor distribution appears to agree only with the ferret experiments, and probably only with H7N9 transmission in ferrets, since avian H5N1 IAVs were not transmissible in ferrets [78, 190]. This conceptual model is apparently an oversimplification. There may be more factors involved in the transmission of IAVs, such as the functional balance between the HA and NA proteins and inherent replicability of IAVs [191, 192]. The problem is, due to the discrepancy between what is expected in humans by the conceptual model and the reality, how to translate the results of transmission experiments evaluated in animal models to the natural transmission environments between humans. We may compare the transmissibility of certain viral isolates to that of CA04 in ferrets, but it is difficult to determine what the level of the transmissibility of these viruses indicate in terms of the pandemic potential of the viruses. The reports on the α-2,3 SA expression status in the upper respiratory tract of ferrets, pigs, and humans appear to be inconclusive [63]. Determination of IAV receptor distributions in humans and animal models appears critical for the interpretation of pathogenicity and transmission experiments assessed in animal models.

### Pandemic potential of MERS-CoV

We have discussed how to approach to the determination of the pandemic potential of IAVs. Similar principles may apply to MERS-CoVs. Previously, MERS-CoV replication was noted in NHP-derived cell lines, Vero, and LLC-MK2 cells [28]. It is now known that DPP4 is a functional cellular receptor for MERS-CoVs and that Vero cells express DPP4 [193]. Vero cells also express α-2,3 SA, which has been shown to assist receptor binding of MERS-CoVs [130, 151, 152, 167]. Vero cells are well known for an impairment in the type I interferon pathways [194]. Hence, Vero cells for MERS-CoV infection may function like MDCK cells for influenza viruses. Growth of MERS-CoVs in Vero cells may indicate their inherent replication potential. Better growths of MERS-CoVs in the upper respiratory tract is likely to contribute to their better transmission [143]. Therefore, growth characteristics of camel MERS-CoVs in Vero cells may give us initial clues about how efficient the transmission of novel strains might be.

### Pathogenicity of MERS-CoVs in hDPP4 mice

The pathogenicity of MERS-CoVs is closely associated with DPP4 expression in the lower respiratory tract of humans [153]. Like IAVs, the pathogenicity of MERS-CoV in hDPP4 mice may be an initial indicator. While hDPP4 mice produced severe symptoms upon MERS-CoV infection [140], mice whose mDPP4 replaced with hDPP4 or modified to contain MERS-CoV binding hDPP4 motif (m-hDPP4-mice) showed little clinical signs [138, 139]. hDPP4 mice might be good for the evaluation of antiviral candidates, but not as a pathogenicity indicator, due to the ectopic expression of hDPP4. hDPP4 mice might be better to observe increases of growths and clinical signs after MERS-CoV infection. Comparison of viral titers in the lungs and the lung pathology of MERS-CoV infection with those of the first MERS-CoV isolate HCoV-EMC [28] would give clues concerning the replication potential of MERS-CoVs.

### Surrogate models for the evaluation of MERS-CoV transmission

There are no small animal models for the evaluation of MERS-CoV transmission yet. Even though avian IAVs transmit extremely poorly between humans, MERS-CoVs appears highly transmissible between humans. In case of MERS-CoV outbreaks in Korea, 2015, a super-spreader individual resulted in 28 infection cases [34]. However, it is difficult to appropriately interpret the transmissibility difference between IAVs and MERS-CoVs in ferrets. Due to the distribution differences of DPP4 and α-2,3 SA in the lower and upper respiratory tracts in humans and dromedary camels, it might be a major problem in translating the results of transmission experiments using small animal models. HCoV-EMC exhibited no transmission in rabbits [143], and a kind of transmission threshold has not been defined, so it would be a problem to determine the pandemic potential of MERS-CoV isolates. The DPP4 motif of NHPs have the same sequence with that of humans [132, 147]. However, no MERS-CoV transmission has been reported between NHPs. Hence, some kinds of surrogate measures might be necessary to evaluate the transmissibility of MERS-CoVs. Mice are not capable of contact or aerosol transmitting IAV but can be infected with aerosolized live-attenuated influenza vaccine virus using a nebulizer [195]. Since it has been reported that the lower respiratory tract of hDPP4 mice is very similar to that of humans, replication kinetics of a MERS-CoV isolate in the lungs of hDPP4 mice would provide a clue concerning transmissibility and virulence of MERS-CoVs. This suggests the feasibility and importance of hDPP4 mice for the evaluation of replication or transmissibility of MERS-CoVs.

## Conclusion

We have discussed what may be required to be a pandemic virus by analyzing the past influenza pandemics. The uniqueness of the past influenza pandemics is in that the three pools of reservoirs or hosts (avian, swine, and human) keep the persistent potential of generating novel IAVs and that no other pathogens are known to bring about pandemics recurrently. Moreover, the ingredients of the influenza pandemics and the modes of transmission may apply to other pathogens exhibiting pandemic potential. Unlike IAVs, MERS-CoVs have not swept global communities and appeared to need a persistent human reservoir. The ultimate goal of MERS-CoV researches may find a way to prevent a MERS-CoV pandemic. Studying the pandemic viruses, such as pdm1918 and pdm2009, may provide scientific information regarding molecular and viral requirements of potential pandemic viruses. Our conceptual interpretation of animal models also underlines the value and importance of preclinical experiments in terms of the translational purposes and insights of the pandemic potential of influenza and other RNA viruses.

## Data Availability

Not applicable.
